# Preoperative hyaluronic acid injection modulates postoperative functional outcome in patients undergoing arthroscopic rotator cuff repair

**DOI:** 10.1186/s13018-020-01715-5

**Published:** 2020-06-03

**Authors:** Yosuke Nakamura, Masafumi Gotoh, Yasuhiro Mitsui, Hidehiro Nakamura, Hiroki Ohzono, Takahiro Okawa, Naoto Shiba

**Affiliations:** 1 Department of Orthopedic Surgery, Kurume University Hospital, 67 Asahi-machi, Kurume, Fukuoka 830-0011 Japan; 2 Department of Orthopedic Surgery, Kurume University Medical Center, 155-1 Kokubu-machi, Kurume, Fukuoka, 839-0863 Japan

**Keywords:** Rotator cuff tear, Arthroscopic rotator cuff repair, Hyaluronic acid, Subacromial injection, Functional outcome, Propensity score analysis

## Abstract

**Background:**

Arthroscopic rotator cuff repair (ARCR) generally yields acceptable clinical results. Hyaluronic acid (HA), a high-molecular-weight polysaccharide, is present in the extracellular matrix of soft connective tissue and synovial fluid, and its injection is known to significantly improve pain and clinical outcomes after rotator cuff injury. Some studies have described the role of HA injections as conservative therapy for rotator cuff tears. Since the subacromial bursa is believed to be the main source of shoulder pain in rotator cuff tears, subacromial injection is frequently used before surgery; however, its relationship with the clinical outcome after surgery remains unclarified. Therefore, we aimed to examine effects of preoperative subacromial HA injection on postoperative clinical outcome in patients with ARCR.

**Methods:**

Ninety-eight patients were divided into a HA injection group and a non-injection group. The functional outcome measured was the University of California, Los Angeles (UCLA) score. Univariate analysis was performed to obtain variables with *p* values less than 0.1; we then used propensity score analysis, adjusting for pre- and post-operative confounding factors.

**Results:**

The UCLA scores of all patients significantly improved 1 year postoperatively (PO) (*p* < 0.05). Subacromial HA injections were performed in patients with worse preoperative function. Univariate analysis showed significantly greater improvements in the injection group than in the non-injection group in terms of preoperative UCLA score, trauma, diabetes mellitus, UCLA score 3 months PO, abduction strength 4 months PO, and internal rotation (IR) strength 6 and 12 months PO. Propensity score analysis demonstrated that UCLA scores 3 months PO and IR strength 12 months PO in the injection group were significantly greater than those in the non-injection group. There were no significant differences in postoperative re-tear rates between the groups. In sub-analysis of the injection group, propensity scores showed that concurrent use of local anesthetics did not affect the data, suggesting that HA was effective.

**Conclusion:**

Subacromial injection was administered to patients with worse function before ARCR. Propensity score analysis successfully demonstrated that functional outcome after surgery was improved in patients who were administered this injection compared with patients who were not administered this injection before surgery.

## Background

Rotator cuff tears cause pain and impaired mobility of the shoulder in middle-aged and older patients. They are often treated by arthroscopic rotator cuff repair (ARCR) surgery. Rouhani et al. reported that preoperative treatment with oral COX-2 inhibitors improves pain in the early postoperative period, thus helping achieve good outcomes after ARCR [1]. However, a study by Inderhaug et al. of postoperative outcomes after 6–9 years found that preoperative nonsteroidal anti-inflammatory drug (NSAID) treatment was a factor that contributed to poor outcomes [2]. Donohue et al. reported that patients who received a steroid injection in the subacromial bursa (SAB) before undergoing ARCR had significantly better visual analog scale (VAS) scores, American shoulder and elbow surgeons (ASES) shoulder scores, and constant scores 1 year postoperatively (PO) than patients who did not receive these injections [3]. Another study found that steroid injections after ARCR reduced pain and improved range of motion 3 months PO compared with a control group [4]. Desai et al. reported a strong correlation between 2 or more steroid injections into the subacromial bursa before rotator cuff repair and the need for further surgery [5]. Cancienne et al. found that intraoperative steroid injections had a major effect on postoperative infection rates [2].

Hyaluronic acid (HA), a high-molecular-weight polysaccharide, is present in the extracellular matrix of soft connective tissue and synovial fluid, playing various physiological roles depending on the tissue. The value of HA’s therapeutic effect is well attested, and there have been no reports of adverse events caused by HA injection. Lim et al. compared the efficacy of steroid injections with that of HA injections for arthritis of the shoulder and found that both brought about significant improvement, with no significant difference between the 2 [6]. Shibata et al. evaluated the use of HA and steroid injections for rotator cuff tears and found that pain and the University of California, Los Angeles (UCLA) scores improved significantly after treatment with either, compared with scores before treatment [7]. In a systematic review, Osti et al. found that intra-articular HA injections significantly improved pain and clinical outcomes after rotator cuff injury [8]. However, although some studies have described the role of HA injections as a conservative therapy for rotator cuff tears, few have evaluated its postoperative effect. We therefore treated rotator cuff tear patients with HA injections before ARCR and investigated its postoperative effects.

## Methods

This retrospective study was approved by the institutional review board of the university where this study was conducted (no. 18136). Due to the retrospective nature of the study, the requirement of patient consent was waived.

### Subjects

A total of 205 patients underwent ARCR at our institution between January 2014 and December 2016. The inclusion criteria required that patients (1) underwent ARCR and (2) complete a 1 year follow-up PO. The exclusion criteria included patients who (1) had systemic disease, (2) had fractures around the shoulder, and (3) underwent preoperative injections with agents other than HA (e.g., steroids). Finally, 98 patients with an average age of 63.5 ± 9.1 years were included. Depending on whether subacromial injection was given before surgery, patients were divided into 2 groups as follows: the injection group (58 patients, 23 patients with HA only, and 35 patients with HA + local anesthetics) and the non-injection group (40 patients).

Injections were given < 5 times in 25 patients and ≥ 5 times in 33 patients; of the 58 patients who received injections, 25 received them in our hospital and 33 at other hospitals. Members of both groups received NSAID therapy (41 patients in the injection group and 30 in the non-injection group) and physiotherapy (58 patients in the injection group and 40 in the non-injection group). The mean duration of preoperative treatment was 8.4 months in the injection group and 8.9 months in the non-injection group.

### Surgical technique and postoperative regimen

Patients underwent ARCR in the beach-chair position under general anesthesia along with an interscalene block. The torn cuff was repaired using the double-row suture bridge technique. For suture bridge repair, 1 row of anchors was placed in the medial aspect of the footprint with or without tying, and transosseous repair of the torn cuff with a knotless anchor on the lateral aspect of the footprint was completed. If needed, additional procedures including capsular release, tenotomy, or tenodesis of the long head of the bicep tendon were performed. Acromioplasty was performed in all cases.

Patients were immobilized using a sling with an abduction pillow postoperatively, with the shoulder internally rotated at 30°–40° and abducted at 20°. Passive range of motion (ROM) exercises of the shoulder commenced on postoperative day 4 for small/medium tears and on day 22 for large/massive tears. For both tear types, active ROM and isotonic muscle strengthening exercises were allowed at postoperative week 6 and postoperative week 12, respectively.

### Outcome measures

The functional outcome measure was the UCLA score. ROM was assessed using a goniometer, and muscle strength was measured using a hand-held dynamometer (Micro FET2; Hoggan Health Industry, West Jordan, UT, USA). VAS scores were reported based on patients’ subjective assessments.

Structural evaluation was performed using magnetic resonance imaging (MRI), according to a previous report [4]. A postoperative “intact tendon” was defined as types I–III in the Sugaya classification [9]. The tear length and width were evaluated as the coronal and sagittal oblique distance on T2-weighted images, respectively [10]. These measures were evaluated preoperatively and at 3 or 6, and 12 months PO.

### Statistical analysis

The JMP12 statistical software (SAS Institute, Cary, NC, USA) was used for statistical analyses. Because of the retrospective study design, the effect of HA injections on postoperative factors was evaluated after adjusting for confounding factors. Specifically, univariate analyses were performed to extract potential confounding factors with *p* values less than 0.1. Then, propensity scores were calculated with these potential confounding factors as explanatory variables. Finally, the propensity score was fitted to evaluate the injections at each follow-up period. Data are expressed as mean values with standard deviations. *P* values < 0.05 were considered to be statistically significant.

## Results

**Fig. 1:**
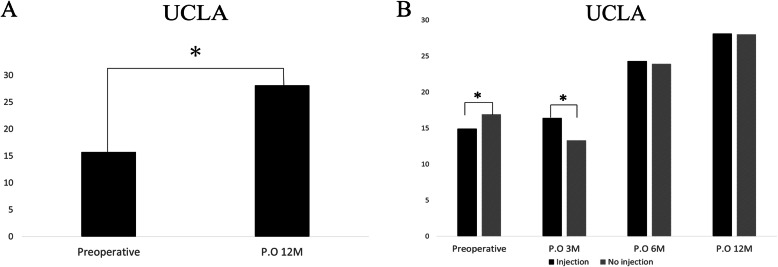
UCLA scores. **a** Before and after surgery in all patients. **b** Comparison of UCLA scores between injection and non-injection groups before and after surgery. UCLA score, University of California, Los Angeles score

UCLA scores of all patients significantly improved from 28.1 points preoperatively to 15.7 points 1 year PO (*p* < 0.05) (Fig. 1a). The mean total UCLA scores were significantly different between the 2 groups preoperatively and 3 months PO (*p* > 0.04 and *p* > 0.03, respectively) (Fig. 1b).

For functional outcomes, univariate analysis showed differences (*p* < 0.1) in the following items: UCLA score at 3 months PO, abduction strength at 4 months PO, and internal rotation (IR) strength 6 and 12 months PO. By contrast, there were no differences in structural outcomes between the groups (Table 1).

**Table 1:** Comparison between injection/non-injection groups by univariate analysis

Functional outcome			
Univariate analysis			
Variables	Injection group	Non-injection group	*p* value
Age (years)	64.4	62.1	0.22
Sex (*n*)			
Male	32 (55%)	28 (70%)	
Female	26 (44%)	12 (30%)	0.13
NSAID (*n*)	37 (63%)	24 (60%)	0.70
Hard work (*n*)	21 (38%)	13 (35%)	0.71
Diabetes mellitus (*n*)	2 (3%)	5 (12%)	0.08^a^
Contracture (*n*)	15 (25%)	7 (17%)	0.32
Trauma (*n*)	27 (56%)	13 (37%)	0.08^a^
Workmen’s accidents (*n*)	8 (15%)	5 (14%)	0.88
Worker’s compensation (*n*)	8 (15%)	5 (14%)	0.88
Follow-up period (months)	8.4	8.9	0.81
Preoperative			
VAS (rest)	2.1	2.1	0.97
VAS (motion)	5.6	4.7	0.14
VAS (night)	4.5	3.6	0.17
Preoperative ROM			
Elevation (°)	107	112	0.52
Abduction (°)	99	106	0.44
Internal rotation (level of intervertebral body)	5.2	5.5	0.62
External rotation (°)	48	45	0.51
Preoperative muscle strength			
Elevation (relative ratio to uninvolved side)	70	75	0.28
Abduction	72	74	0.77
Internal rotation	72	85	0.58
External rotation	90	87	0.17
UCLA score	14	16	0.04^a^
Postoperative (PO) VAS			
PO 3 M VAS (rest)	0.6	0.7	0.86
PO 3 M VAS (motion)	1.4	2.2	0.04^a^
PO 3 M VAS (night)	0.8	1.4	0.06^a^
PO 4 M VAS (rest)	0.4	0.7	0.19
PO 4 M VAS (motion)	2.1	2.1	0.88
PO 4 M VAS (night)	1.0	1.5	0.18
PO 6 M VAS (rest)	0.3	0.6	0.11
PO 6 M VAS (motion)	1.5	1.9	0.24
PO 6 M VAS (night)	0.7	0.9	0.55
PO 12 M VAS (rest)	0.3	0.4	0.61
PO 12 M VAS (motion)	1.1	1.0	0.59
PO 12 M VAS (night)	0.4	0.3	0.62
Postoperative ROM			
PO 3 M elevation (°)	97	89	0.32
PO 3 M abduction (°)	83	79	0.68
PO 3 M internal rotation (level of intervertebral body)	2.9	3.1	0.71
PO 3 M external rotation (°)	20	17	0.48
PO 4 M elevation (°)	114	119	0.44
PO 4 M abduction (°)	104	112	0.38
PO 4 M internal rotation (level of intervertebral body)	4.8	4.3	0.44
PO 4 M external rotation (°)	28	28	0.93
PO 6 M elevation (°)	134	126	0.24
PO 6 M abduction (°)	133	127	0.39
PO 6 M internal rotation (level of intervertebral body)	6.6	5.5	0.08^a^
PO 6 M external rotation (°)	36	34	0.57
PO 12 M elevation (°)	140	141	0.79
PO 12 M abduction (°)	142	143	0.88
PO 12 M internal rotation (level of intervertebral body)	8.1	7.9	0.80
PO 12 M external rotation (°)	38	38	0.87
Postoperative (PO) muscle strength			
PO 3 M elevation (relative ratio to uninvolved side)	33	31	0.68
PO 3 M abduction	34	31	0.47
PO 3 M internal rotation	71	67	0.53
PO 3 M external rotation	58	57	0.95
PO 4 M elevation (relative ratio to uninvolved side)	55	48	0.25
PO 4 M abduction	61	49	0.06^a^
PO 4 M internal rotation	82	80	0.70
PO 4 M external rotation	77	74	0.82
PO 6 M elevation (relative ratio to uninvolved side)	70	59	0.10
PO 6 M abduction	71	64	0.39
PO 6 M internal rotation	90	93	0.58
PO 6 M external rotation	81	76	0.57
PO 12 M elevation (relative ratio to uninvolved side)	82	78	0.60
PO 12 M abduction	78	77	0.85
PO 12 M internal rotation	100	90	0.06^a^
PO 12 M external rotation	83	87	0.65
Postoperative UCLA score			
PO 3 M UCLA score	16	13	0.03^a^
PO 4 M UCLA score	20	18	0.41
PO 6 M UCLA score	24	23	0.74
PO 12 M UCLA score	28	28	0.93

Data were evaluated by logistic analysis

*NSAID* non-steroidal anti-inflammatory drugs, *ROM* range of motion, *VAS* visual analog scale, *UCLA score* University of California at Los Angeles score

^a^*p* value < 0.1

Next, propensity score analysis was performed to adjust for the confounding factors identified by univariate analysis. The results demonstrated that UCLA scores 3 months PO and IR strength 12 months PO were significantly better in the Injection group than in the non-injection group (Table 2). In the same manner, propensity score in sub-analysis in the injection group showed that co-use of local anesthetics did not affect the data, suggesting that the HA injections were effective.

**Table 2:** Comparison between injection and non-injection groups after adjustment by propensity score analysis

	Injection	Non-injection	*p* value
UCLA score at 3 months	16.4	13.3	0.03
IR strength at 12 months	1.0	0.9	0.02

*UCLA score* University of California at Los Angeles score, *IR* internal rotation

## Discussion

Our previous studies confirmed the various positive effects of HA injections on rotator cuff tears. In subacromial synovial fibroblasts, HA inhibited inflammatory cytokine production via the CD44 receptor; moreover, in glenohumeral synovial fibroblasts, HA inhibited adhesion-induced cytokine production via the CD44 receptor [11]. Honda et al. reported that HA may accelerate fibrocartilage formation in tendon-to-bone healing, with enhancement of this biomechanical property [12].

Several studies have reported the benefits of conservative therapy. Shibata et al. evaluated the effect of HA and steroid injections for rotator cuff tears after 4 weeks and 24 weeks, and found that pain and UCLA scores improved significantly after treatment with either injection [7]. In a randomized controlled trial, Blaine et al. reported that HA injections in the shoulder significantly alleviated pain, particularly at night [13]. Osti reported that intra-articular HA injections were effective in reducing pain and improving function in shoulders with rotator cuff tears, without severe adverse reactions [8]. Nevertheless, no studies have evaluated the effects of HA on clinical outcomes in patients with rotator cuff repairs. The present study compared the postoperative course in patients who underwent HA injections prior to surgery with those who did not received injections. We found that HA injections accelerated functional outcomes in terms of UCLA score 3 months PO and IR strength 12 months PO.

Despite having been worse preoperatively, UCLA scores improved postoperatively in the injection group. Many of the subjects had been referred to our hospital before surgery (of the 58 patients in the injection group, 25 received injections at our hospital and 33 at another hospital), and their clinical assessments at the start of treatment were unknown. However, the fact that preoperative UCLA scores were significantly lower in patients in the injection group suggested that their symptoms may have been relatively severe. Nevertheless, the fact that UCLA scores 3 months PO were significantly higher both before and after propensity score analysis correction provided supporting evidence for the effectiveness of HA injections.

Some patients in the injection group also received local anesthetic injections. Local anesthetics are cytotoxic to chondrocytes and tenocytes. Single-dose intra-articular administration of local anesthetics impedes chondrocyte metabolism and should be performed at low concentrations only for selected diagnostic purposes and painful joints [9]. Honda et al. reported that, in a rotator cuff tear model, local anesthetics led to apoptosis of tenocytes and delay of the granulation tissue maturation, causing biomechanical weakness of the enthesis involved [14]. Lee et al. reported that local anesthetic injections into the SAB or shoulder joint immediately after ARCR significantly improved pain up to 24 h PO [15] Cook et al. compared SAB steroid or local anesthetic injections for rotator cuff-related shoulder pain and found that steroid injections improved pain significantly more effectively for up to 8 weeks; however, after this point, there was no difference between the 2 groups [16], and no negative effects have been reported in clinical practice. In a sub-analysis comparing HA injections alone with the combination of HA and local anesthetic injections, we found that there were no differences between the 2 in terms of either functional or imaging findings. This suggested that the combined use of local anesthetic had no effect on the efficacy of HA, including any toxic action.

Another common treatment for rotator cuff tears in addition to HA is steroid injections. Donohue et al. reported that patients who received a steroid injection in the SAB before undergoing ARCR had significantly better VAS scores, ASES shoulder scores, and constant scores 1 year PO compared with patients who did not receive these injections [16]. In a study of 12,000 patients, Desai et al. found that 2 or more steroid injections into the SAB before rotator cuff repair surgery significantly increased the risk of requiring further surgery within 2 years PO [5]. Basic research has also shown that the negative effects of steroids include inducing tenocyte apoptosis [17]. Reports on steroids are therefore divided; nevertheless, as far as we are aware, no adverse effects of HA injections have ever been reported. Lim et al. compared the efficacy of steroid injections with that of HA injections in the shoulder joint for shoulder periarthritis after 12 weeks, and found that both significantly improved pain, ASES shoulder score, and constant score, with no significant difference between the 2 [14]. Randomized controlled trials and further studies to investigate the effectiveness of HA and steroid injections are required in the future. Limitations of this study was retrospective, had a small sample size, was short-term, and the follow-up rate was low. Nevertheless, by compensating for these limitations using propensity scores, we successfully demonstrated the positive effects of HA on functional recovery after ARCR.

## Conclusions

We used propensity score analysis to investigate the effect of HA injections into the SAB on postoperative function prior to ARCR. We found that preoperative HA injections significantly improved UCLA score 3 months PO and IR strength 12 months PO.

## Data Availability

Data sharing is not applicable to this article as no datasets were generated or analyzed during the current study.

## Abbreviations

ARCR: Arthroscopic rotator cuff repair
NSAID: Nonsteroidal anti-inflammatory drug
SAB: Subacromial bursa
VAS: Visual analog scale
ASES: American shoulder and elbow surgeons
PO: Postoperatively
HA: Hyaluronic acid
UCLA: University of California, Los Angeles
ROM: Range of motion
